# Oncological allograft failure for humerus reconstruction: the ‘strategic’ vascularized fibula

**DOI:** 10.1080/23320885.2025.2572833

**Published:** 2025-10-21

**Authors:** Alessia Pagnotta, Virginia Maria Formica, Stefano Gumina, Francesca Romana Grippaudo, Carmine Zoccali

**Affiliations:** a Hand and Microsurgery Unit, Jewish Hospital of Rome, Rome, Italy; b Oncological Orthopaedics Department, IRCCS - Regina Elena National Cancer Institute, Rome, Italy; c Orthopaedic and Traumatology Unit, Department of General Surgery, Plastic Surgery, Orthopedics, Policlinico Umberto I Hospital-Sapienza, University of Rome, Rome, Italy; d Department of Plastic Surgery, Policlinico Umberto I, Sapienza University of Rome, Rome, Italy

**Keywords:** Free vascularized fibula graft, microsurgery, post-oncological reconstruction, humerus reconstruction

## Abstract

The allograft for humeral reconstruction is a widely used technique in oncology but complication rates are high and Free Vascularized Fibula Graft (FVFG) represents a “strategic” solution to preserve satisfactory upper limb function. We treated 3 cancer patients with allograft failures and FVFG was used to reconstruct complex humeral defects.

## Introduction

Humerus sarcomas commonly affect young and functionally active individuals, necessitating a safe and reliable reconstruction of the arm [1]. The primary goals of reconstruction include restoring joint integrity, bridging diaphyseal defects, restoring limb function, and enhancing quality of life [2].

For resections involving joints, megaprostheses are typically considered the gold standard, while allograft-prosthetic composites may be suitable for high-demand patients with low-grade tumors. However, the use of articular allografts must be carefully evaluated due to their high rate of mechanical failure.

In cases of intercalary resections, reconstructions can be accomplished using an intercalary prosthesis; however, a significant incidence of mechanical complications has been documented. Consequently, both vascularized and non-vascularized biological reconstructions are often preferred to achieve a more secure outcome. While Free Vascularized Bone Grafts (FVBG) are considered the optimal choice, allografts may also provide satisfactory outcomes, especially in cases involving small bone gaps, patients with lower functional demands, or when a less invasive surgical approach is recommended to facilitate the prompt initiation of medical treatments.

Nevertheless, allograft possible complications must be carefully considered. These include non-union, infection, and mid- to long-term issues such as hardware failure and allograft fracture, all of which should be considered in light of the patient’s specific needs [3–5].

These complications are more prevalent in patients undergoing chemotherapy and/or radiotherapy [5].

Mechanical failures seen in allograft reconstructions are frequently linked to allograft necrosis and resorption, prompting the consideration of secondary Free Vascularized Bone Grafts (FVBG) as a viable option to rejuvenate the affected segment. Possible donor sites for FVBG include the fibula, iliac crest, medial femoral condyle, and ribs [6,7].

Vascularized free fibula graft (FVFG) is commonly favored for humeral reconstructions, especially when addressing extensive bone defects. Furthermore, FVFG is a preferred option in cases involving poorly vascularized or scarred soft tissue, including those resulting from chemotherapy and radiotherapy treatments.

Custom-made reconstructions using 3D printing are an emerging approach in the treatment of bone defects [8], and hybrid constructs using 3D prostheses and vascularized fibula have been described [9].

The objective of this study is to present three illustrative and difficult cases in which FVFG was employed to restore humeral continuity following mechanical failure in functionally active patients. These individuals had previously undergone intercalary resection and reconstruction with allograft, plates, and screws for the treatment of humeral bone tumors.

## Patients/material and methods

Between February 2015 and April 2018, at the Jewish Hospital of Rome, we managed three cases of humeral allograft failure following oncological reconstruction utilizing FVFG (Table 1). Written informed consent has been obtained from the patient to publish this paper. Each patient presented with allograft nonunion accompanied by either hardware failure or loosening. Pre-operative assessments encompassed ultrasound color Doppler imaging of both upper and lower limbs, X-rays, computed tomography (CT) scans, magnetic resonance imaging (MRI), and CT-scan, MRI, and PET-scan.

**Table 1.** Patients characteristics.

Case	Tumor Type	Age	Sex	1st surgery and complication	Bone Gap	FVFG Length	Anastomosys	Healing Time	Complications
1	Pleomorphic Lyposarcoma	48 y.o	Male	Cadaver diaphysis allograft. Nonunion with hardware failure (plate loosening)	12cm	17cm	Fibular A/V – ulnar recurrent A/V	10 months	None
2	G1 Chondrosarcoma	47 y.o	Male	Cadaver metaphysis allograft. Nonunion with hardware failure (plate breakage)	2cm	14cm	Fibular A/V – profunda brachii artery A/V	10 months	None
3	Dedifferenziated Chondrosarcoma	76 y.o	Male	Cadaver diaphysis allograft. Nonunion with hardware failure (2 screws breakage and plate loosening)	15cm	22cm	Fibular A/V – ulnar recurrent A/V	12 months	-Surgical wound dehiscence -Moderate lymphedema of lower limb -Fracture of fibula diaphysis caused by trauma

Data from three patients treated for humeral allograft failure following oncologic reconstruction between February 2015 to April 2018.

### Case 1

A 47-year-old, right-hand dominant patient was diagnosed with high-grade pleomorphic liposarcoma of the proximal humerus, with sparing of the humeral head (Table 1). The patient underwent wide tumor resection followed by reconstruction with an intercalary allograft, secured with screws and plating. The decision to use an allograft as primary reconstruction was driven by the urgency to begin medical therapy, given the diagnosis of aggressive high-grade sarcoma. Preoperative staging revealed no evidence of metastatic lesions. Postoperatively, the treatment regimen included both chemotherapy and radiotherapy.

Eleven months after surgery, the patient presented with pain and deformity in the humerus. Radiographic evaluation revealed non-union between the allograft and the native humerus, as well as loosening of the fixation hardware.

Considering the patient’s characteristics, including age and activity level, as well as the integrity of the axillary nerve and deltoid muscle function, a treatment plan was devised involving partial removal of the allograft, reconstruction with a FVFG, and new osteosynthesis. Following bone debridement and revitalization of the residual bone segments, the FVFG was carefully positioned at the recipient site and fixed with plates and screws. The fibular vessels were anastomosed to the ulnar recurrent artery and vein to ensure adequate vascularization.

Ten months after surgery, radiographic imaging confirmed successful bone fusion, and the patient demonstrated a satisfactory functional outcome. However, at 16 months, the patient developed metastasis at the eighth thoracic vertebra (D8) with resultant paraplegia. Despite this complication, the patient retained the ability to use his reconstructed right arm for ambulation with crutches. Tragically, 25 months after the surgery, the patient died due to systemic disease progression.

### Case 2

A 47-year-old, right-handed male was diagnosed with Grade 1 (G1) chondrosarcoma of the left humeral head (Table 1). Due to the lesion’s specific anatomical location, a navigation-guided resection of the proximal humeral metaphysis was performed with preservation the articular surface. Reconstruction was achieved using a matched structural allograft to restore the shape of the metaphyseal region, secured with a plate and screws. The decision to use an allograft as primary reconstruction was motivated by the need to reconstruct the shape of the metaphysis with rotator cuff insertion.

**Figure 1.**
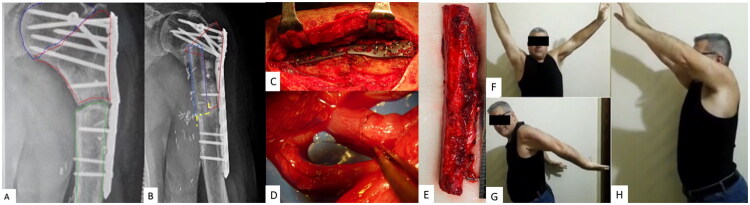
(Case 1). A. X-ray after first surgery showing oncological resection, reconstruction with intercalary allograft and broken plate. Humeral head is surrounded by blue dashed line. Intercalary allograft is surrounded by red dashed line. Humeral shaft is surrounded by green dashed line. B. X-ray showing radiological healing of the hybrid procedure at 10 months after second surgery. Intercalary allograft is surrounded by red line. FVFG is surrounded by blue dashed line. Step-cut osteotomy is underlined by yellow dashed line. C. Intraoperative picture of the broken plate. D, E. Microvascular anastomosis and FVFG. F, G, H. Clinical pictures of the patient with satisfactory range of motion of the left arm at 12 months after surgery.

At eleven months postoperatively, radiographic evaluation revealed non-union at the distal allograft-bone interface, accompanied by hardware failure (Figure 1(A,C)). This complication was attributed to excessive joint activity, contrary to postoperative restrictions advised by the orthopedic team. Notably, successful fusion had occurred at the proximal allograft-bone interface. In light of partial union, a hybrid reconstruction technique was undertaken to address the failed segment (Figure 1(B)). A 14 cm FVFG (Figure 1(E)) was proximally inserted into the existing allograft, while distally it was positioned in a step-cut osteotomy created in the residual proximal humeral diaphysis.

Microvascular anastomoses were performed between the fibular vessels and the profunda brachii artery and vein (Figure 1(D)). Ten months postoperatively, radiographic evaluation demonstrated successful bone healing, and the clinical outcome was considered satisfactory (Figure 1(F–H)). After 6 years, the patient remains disease-free and has resumed recreational activities, including archery.

### Case 3

**Figure 2.**
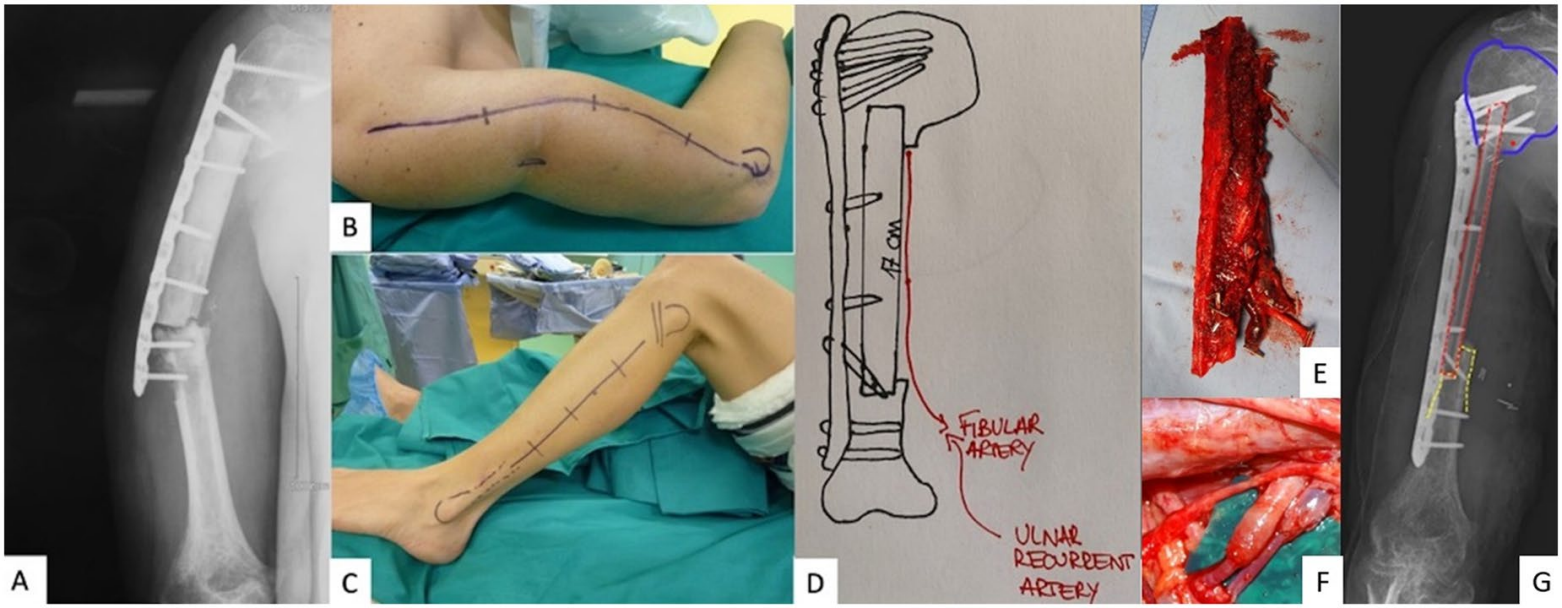
(Case 2). A. X-ray exams 11 months after surgery showing allograft non-union and hardware breakage. B. Clinical deformity of the right arm. C. Clinical picture of donor site for FVFG harvesting. D. Image of preoperative planning. E. FVFG harvested with peroneal vessels. F. Microvascular anastomosis between peroneal vessels and ulnar recurrent vessels. G. Postoperative X-ray. The humeral head is surrounded by blue dashed line. FVFG is surrounded by red dashed line. Step-cut osteotomy is underlined by yellow dashed line.

A 76-year-old, amateur skier, right-hand dominant male, presented with dedifferentiated chondrosarcoma involving the diaphysis of the right humerus. He underwent a 15-cm intercalary resection, followed by reconstruction with structural allograft stabilized with a plate and screws (Table 1). The decision to use an allograft for primary reconstruction was based on the patient’s age, under the assumption of low functional demand. However, at 12 months postoperatively, radiographic imaging indicated non-union and hardware failure (Figure 2(A–B)). Consequently, the decision was made to remove the allograft, and a 22 cm FVFG was harvested (Figure 2(C–E)) and secured with a plate and screws. The fibular vessels were anastomosed to the ulnar recurrent artery and vein (Figure 2(F)).

The patient experienced two minor complications: surgical wound dehiscence and moderate lower limb lymphedema, both of which resolved within a few months. Radiographs taken 12 months after the final surgery demonstrated successful bone healing and the clinical outcome was considered satisfactory (Figure 2(G)).

Five years after surgery, the patient sustained a fracture of the diaphyseal portion of the fibula accompanied by breakage of three distal screws during a skiing accident. Radiographic evaluation confirmed union at the proximal and distal interfaces, but further investigations at that time revealed evidence of systemic disease progression.

## Results

The mean age of the patients at the time of surgery was 58.3 years. Graft fixation was achieved using locking plates and screws in all cases. Two of the three patients were followed clinically and radiologically for five and six years postoperatively. One patient died from multiorgan metastases two years after surgery. Bone union was achieved at a mean of 10.6 months.

## Discussion

The main indication for free vascularized bone grafts (FVBG) is bone defects that are over 6 cm in length, or under 6 cm but considered ‘critical’ (due to poorly vascularized tissues, radiation treatment, chemotherapy, prior surgery, or previous infection) [10]. A vascularized bone is crucial for speeding up the bone-healing process and preventing infection, as well as improving biomechanical and functional results [11]. The surgical technique is complex and requires trained microsurgeons. For this reason, allograft reconstructions are sometimes preferred from an oncological perspective, as they allow for a technically simpler surgery and faster recovery. Nevertheless, complications are known to be influenced by a variety of factors. These include the specific anatomical segment involved—with the humerus been associated with particularly high complication rates—as well as patient age, smoking habits, allograft length and contact area, fixation techniques, and the use of non-bridging osteosynthesis. These factors collectively contribute to the overall risk profile and outcomes associated with such surgical procedures [12]. Non-union and hardware failure represent the principal indications for revision surgery, and in active patients the FVFG emerges as a reliable and effective strategy to address these complications. When placed in the recipient site, the fibula undergoes a remodeling process that facilitates proper functionality. Consequently, radiographic assessments often reveal the development of progressive hypertrophy, indicating successful integration and adaptation of the graft [13,14].

In our view, the intercalary positioning of the fibula is of paramount importance. Maximizing the contact surface between the allograft and the patient’s bone is a key strategy to promote successful osseous fusion. The use of a step-cut osteotomy technique effectively increases the contact area, thereby facilitating graft integration and bone healing.

The placement of the fibula was determined by several factors, such as the location of the defect and the potential for achieving union with the existing allograft. Cases 1 and 3 necessitated complete removal of the allograft due to non-union at both the proximal and distal ends. In these instances, the fibula was introduced proximally into the humeral head to enhance contact and facilitate union. Conversely, in case 2, successful union was observed at the proximal aspect of the allograft, allowing for a side-to-side hybrid technique in which the fibula was inserted through the existing allograft.

An additional challenge during these surgical procedures involved the complexity and compromised condition of the recipient tissues. In many instances, these tissues have been exposed to radiotherapy or chemotherapy, and may exhibit scar tissue resulting from prior surgeries. As a result, these regions are particularly fragile, and selecting suitable vessels for the anastomosis can be challenging. Notably, in two out of the three cases presented, the ulnar recurrent artery was utilized for this purpose.

To enhance graft stability, intraoperative fixation was performed using bridging locking plate. In our series, all patients achieved radiological union within a mean period of 10.6 months.

In our opinion, FVFG should be considered the optimal approach for primary oncologic reconstruction of the humerus when addressing large bone defects. Moreover, FVFG represents the only viable reconstructive option in cases of allograft failure when preservation of limb function remains a priority. The use of an allografts may be appropriate only for patients with small bone gaps and low functional demands.

## Conclusion

Failures of humeral intercalary allografts pose a significant challenge. The utilization of FVFG proves to be a viable solution in such cases, ultimately ensuring successful limb salvage.

## Data Availability

The data that support the findings of this study are available within the article.
